# MWCNT-Au-Pt hybrid nanocomposite–based electrochemical immunosensor for FGF-2 detection: a novel strategy for anxiety disorder diagnosis

**DOI:** 10.1007/s00216-026-06346-z

**Published:** 2026-02-09

**Authors:** Nil Su Çaylayik, Vasfiye Hazal Özyurt, Burak Ekrem Çitil, Ülkü Anik

**Affiliations:** 1 Faculty of Science, Chemistry Department, Mugla Sitki Kocman University, 48000 Kotekli, Mugla Turkey; 2 Research Laboratory Center, Sensors, Biosensors and Nano-Diagnostic Systems Laboratory, Mugla Sitki Kocman University, Kotekli, Mugla Turkey; 3 Faculty of Tourism, Department of Gastronomy and Culinary Arts, Mugla Sitki Kocman University, Kotekli, Mugla Turkey; 4 Faculty of Medicine, Department of Medical Microbiology, Mugla Sitki Kocman University, Kotekli, Mugla Turkey

**Keywords:** Fibroblast growth factor-2, Anxiety disorder biomarker, Human saliva, Hybrid nanomaterials, Immunosensor

## Abstract

The accurate diagnosis of anxiety disorders remains challenging, as current detection methods primarily rely on subjective questionnaires. In this study, a novel electrochemical biosensor for the detection of fibroblast growth factor-2 (FGF-2), a biomarker associated with anxiety disorders, was developed for the first time. The sensing principle was based on monitoring the impedimetric signal changes generated by the interaction between anti-FGF-2 and the FGF-2 antigen, using a carbon screen–printed electrode as the transducer. To enhance the sensitivity and overall performance of the biosensor, a multi-walled carbon nanotube-gold-platinum (MWCNT-Au-Pt) hybrid nanocomposite was incorporated into the immunosensor structure. Critical parameters—including the amount of MWCNT-Au-Pt, the concentration, incubation time, and temperature of anti-FGF-2, as well as the incubation time and temperature for the anti-FGF-2/FGF-2 interaction—were systematically optimized. Under optimized conditions, the developed impedimetric FGF-2 immunosensor exhibited a linear response in the range of 10–100 ng/mL, with a limit of detection of 1.01 ng/mL and a limit of quantification of 3.10 ng/mL. The specificity of the sensor was confirmed in the presence of potential interfering substances. For real sample analysis, human saliva samples (*n* = 30) were diluted appropriately and spiked with FGF-2 at concentrations of 20, 50, and 75 ng/mL. The obtained recovery values demonstrated that the developed impedimetric FGF-2 immunosensor is highly suitable for real-sample applications and holds significant potential as a reliable tool for the detection of anxiety disorders.

**Graphical abstract:**

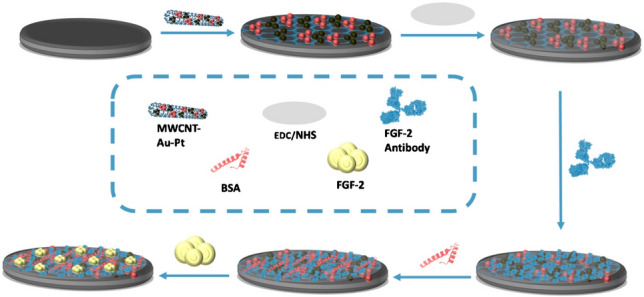

**Supplementary Information:**

The online version contains supplementary material available at 10.1007/s00216-026-06346-z.

## Introduction

Anxiety disorders are among the most commonly reported mental health conditions [1, 2], with global prevalence rates ranging from 3.8 to 25% [2]. Due to their widespread occurrence, these disorders contribute significantly to healthcare costs in many countries. Stress and anxiety trigger physiological changes by altering hormone levels, primarily through the activation of the hypothalamic-pituitary-adrenal axis and the autonomic nervous system—an effect particularly pronounced in chronic anxiety cases [3, 4].

The severity and manifestation of stress responses vary greatly among individuals, influenced by a range of personal and environmental factors. This variability complicates the diagnosis of anxiety disorders and the establishment of clear clinical thresholds [1, 5]. For instance, studies have shown that up to 50% of individuals with anxiety disorders go undiagnosed [6], highlighting the limitations of current diagnostic tools, particularly self-report questionnaires, which often fail to yield accurate results.

Despite ongoing efforts, the underdiagnosis of anxiety disorders and the challenges it poses for effective treatment remain significant issues [7]. As a result, there is a critical need to identify reliable biomarkers involved in the pathogenesis of anxiety disorders to enable earlier, more accurate, and more effective diagnosis and intervention [7, 8].

Fibroblast growth factor-2 (FGF-2) has recently been identified as a potential biomarker for anxiety disorders [9]. FGF-2 is part of a structurally related family of proteins that play a regulatory role in the central nervous system. Members of the FGF-2 protein family are known to influence cell growth, differentiation, migration, and survival across various cell types.

In addition to these roles, FGF-2 has been reported to exert complex effects on both anxiety- and depression-related behaviors [9, 10]. Following the discovery that FGF-2 gene expression is downregulated in individuals with major depression, Turner and colleagues [10] also demonstrated that hippocampal FGF-2 expression was similarly reduced in selectively bred rats with high levels of spontaneous anxiety compared to those with low anxiety. Moreover, knocking down FGF-2 activity in the hippocampus led to increased anxiety in outbred rats. Conversely, administering FGF-2 reduced anxiety levels in highly anxious rats, suggesting that FGF-2 may represent a novel anxiolytic agent [9]. Notably, a study involving a large group of healthy individuals exposed to stressors found a negative correlation between salivary FGF-2 levels and fear expression [8, 11]. These findings suggest that FGF-2 could serve as a promising biomarker for the diagnosis of anxiety disorders.

While the literature primarily reports label-free surface plasmon resonance techniques for FGF-2 detection [12], there is currently no electrochemical biosensor specifically designed for FGF-2. Although electrochemical sensors have been reported for related biomarkers such as FGF-4 [13] and FGF-21 [14], a gap remains for FGF-2-specific electrochemical detection.

Electrochemical biosensors are widely preferred for their practicality, low cost, and high accuracy; meanwhile, nanomaterials play important roles in enhancing sensor performance in terms of sensitivity and accuracy. Among them, carbon-based nanomaterials such as multi-walled carbon nanotubes (MWCNTs) constitute an important class. Moreover, to further improve this enhancement and provide a synergistic effect, modification of these nanomaterials is commonly employed. For example, in this work, MWCNTs were modified with gold (Au) and platinum (Pt) nanoparticles to increase biocompatibility and accelerate electron-transfer kinetics [15]. In this study, an impedimetric FGF-2 immunosensor modified with multi-walled carbon nanotubes-gold-platinum hybrid nanomaterial (MWCNT-Au-Pt) was developed. The incorporation of MWCNT-Au-Pt hybrid nanomaterials into the sensor design enhances sensitivity, making it suitable for sensitive detection of FGF-2 in saliva—a promising approach for anxiety disorder diagnosis. Given the need for reliable, sensitive, and non-invasive diagnostic tools for anxiety disorders, this sensor design offers a robust and practical solution. Following the optimization of key experimental parameters, the analytical performance of the developed MWCNT-Au-Pt-based FGF-2 sensor was evaluated. The optimized system was then successfully applied to 30 diluted saliva samples collected from healthy individuals.

## Experimental

### Materials

Alpha fetoprotein (AFP), bovine serum albumin (BSA), carcinoembryonic antigen (CEA), chloroplatinic acid hexahydrate (H_2_PtCl_6_·6H_2_O), gold(III) chloride hydrate (HAuCl_4_·xH_2_O), hydrogen peroxide (H_2_O_2_), MWCNT, nitric acid (HNO_3_), potassium dihydrogen phosphate (KH_2_PO_4_), potassium permanganate (KMnO_4_), sodium chloride (NaCl), sodium nitrate (NaNO_3_), sulphuric acid (H_2_SO_4_), and trisodium citrate (TSC) dihydrate were purchased from Sigma-Aldrich to prepare hybrid nanomaterials. Potassium dihydrogen phosphate (KH₂PO₄) and sodium hydroxide (NaOH) were purchased from Merck. Potassium ferricyanide (K₃[Fe(CN)₆]) and potassium ferrocyanide (K₄[Fe(CN)₆]), along with N-hydroxysuccinimide (NHS) and N-(3-dimethylaminopropyl)-N′-ethylcarbodiimide (EDC), were obtained from Sigma-Aldrich. The FGF-2 antibody (anti-FGF-2) was supplied by Santa Cruz Biotechnology (HY-P7004), and recombinant bFGF protein (FGF-2) was sourced from MedChemExpress (sc-74412). Carbon screen–printed electrodes (cSPEs) were acquired from Dropsens. All chemicals and reagents used in the experiments were of analytical grade and utilized without further purification.

### Instrumentation

A commercially available cSPE was selected as the transducing element for the constructed FGF-2 biosensor. This electrode comprised a 4-mm-diameter carbon working electrode, accompanied by a carbon counter electrode and a silver reference electrode, all integrated onto a single substrate. Electrochemical impedance spectroscopy (EIS) and cyclic voltammetry (CV) measurements were carried out using a μ-AUTOLAB potentiostat system, supported by NOVA 2.10.8 software and the FRA-2 frequency response module. Surface characterization via atomic force microscopy (AFM) was performed in tapping mode, operating at resonance frequencies ranging between 15 and 29 kHz. All AFM images were captured in ambient air conditions. Prior to scanning electron microscopy (SEM) analysis, samples were coated with a 9-nm-thick gold/palladium (Au/Pd) alloy layer using a Leica EM ACE600 sputter coater. SEM imaging was conducted at a chamber pressure of 1.00–3 Pa, providing a resolution of 1 nm under an accelerating voltage of 1 kV. Infrared spectral data were obtained using a Nicolet iS50 Fourier transform infrared (FTIR) spectrometer (Thermo Scientific Inc.) in attenuated total reflectance (ATR) mode, covering a spectral range from 1 to 4000 cm⁻^1^. Additional characterization studies like X-ray diffractometry (XRD, Philips X’Pert Pro, Tokyo, Japan) and thermo-gravimetric analysis (TGA, TA Instruments SDT Q600) were also carried out, respectively. Finally, all incubation procedures were performed using a BIOSAN ES-20 environmental shaker incubator.

### Procedures

#### Synthesis of MWCNT-Au-Pt hybrid nanomaterial

The MWCNT-Au-Pt nanomaterial was synthesized with slight modifications based on previously reported protocols [15]. The process involved three main stages: activation of MWCNTs, synthesis of gold nanoparticles (AuNPs), and synthesis of platinum nanoparticles (PtNPs), followed by their combination.

In the first stage, MWCNTs were chemically activated. For this purpose, the MWCNTs were sonicated in a strongly acidic mixture composed of 75% (v/v) H₂SO₄ and 25% (v/v) HNO₃ for 6 h. After treatment, the suspension was subjected to multiple centrifugation cycles using ultrapure water to remove residual acids. The collected solid phase was then dried in an oven at 60 °C overnight.

In the second step, AuNPs were synthesized by the reduction of chloroauric acid. A 0.01% (w/w) aqueous solution of HAuCl₄ was brought to a boil, and subsequently, a 1% (w/w) TSC solution was added dropwise while stirring. The reaction mixture was maintained under stirring for 20 min and then allowed to cool to ambient temperature.

The third step involved the preparation of PtNPs. In brief, 0.4 mL of a 5% (w/w) H₂PtCl₆·6H₂O solution was diluted with 34 mL of ultrapure water and heated to 80 °C. While maintaining this temperature, 1% (w/w) TSC solution was slowly introduced, and the mixture was stirred continuously for 4 h.

To fabricate the MWCNT-Au-Pt composite material, 10 mg of activated MWCNTs was dispersed in 5 mL of the previously prepared AuNP suspension and 5 mL of PtNP suspension. The resulting mixture was sonicated for 4 h to ensure uniform distribution of nanoparticles on the nanotube surface. Finally, the nanohybrid was dried at 60 °C overnight to obtain a stable powder form.

#### Preparation of impedimetric FGF-2 immunosensor

In the initial phase of FGF-2 biosensor fabrication, 6 μL of a 6 mg/mL MWCNT-Au-Pt hybrid suspension was carefully deposited onto the surface of cSPE. The modified electrode (MWCNT-Au-Pt@cSPE) was subsequently subjected to incubation at 25 °C for 15 min to facilitate solvent evaporation and ensure uniform surface distribution. Thereafter, 10 μL of 100 mM EDC and 10 μL of 150 mM NHS were sequentially applied to the electrode to activate carboxyl functionalities through carbodiimide chemistry. The functionalized surface was maintained at 25 °C for an additional 15 min to complete the coupling reaction (EDC/NHS@MWCNT-Au-Pt@cSPE).

Following this step, 10 μL of anti-FGF-2 (0.2 μg/mL in phosphate buffer, PBS, pH 7.0) was introduced onto the surface and incubated at 4 °C for 60 min to facilitate covalent attachment via amide bond formation (anti-FGF-2@EDC/NHS@MWCNT-Au-Pt@cSPE). Subsequently, nonspecific adsorption sites were passivated by the addition of 10 μL of 0.1% BSA solution, followed by drying at 25 °C for 15 min to stabilize the blocking layer (BSA@anti-FGF-2@EDC/NHS@MWCNT-Au-Pt@cSPE).

**Scheme 1:**
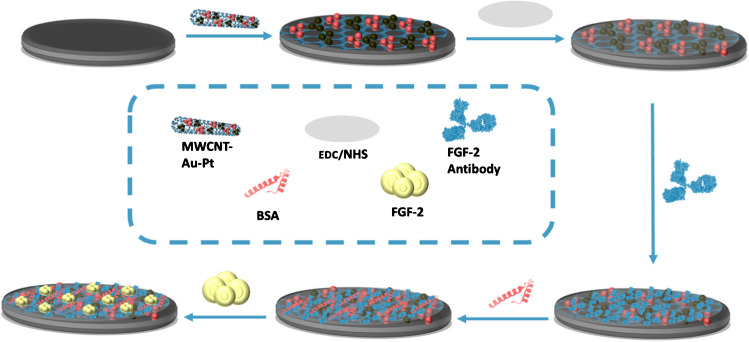
Fabrication of impedimetric FGF-2 immunosensor

In the final fabrication step, 10 μL of varying concentrations of FGF-2 protein (ranging from 1 to 100 ng/mL) was immobilized onto the BSA-coated electrode surface, and the system was incubated at 37 °C for 30 min to allow specific binding interactions (FGF-2@BSA@anti-FGF-2@EDC/NHS@MWCNT-Au-Pt@cSPE). FGF-2 protein and BSA were prepared in phosphate buffer. At each modification stage and prior to EIS measurements, the electrode surface was thoroughly rinsed with 200 μL of 0.1 M phosphate buffer to eliminate unbound molecules. Each electrode was freshly prepared prior to use and employed in a single-use format except for the real sample applications (as showed in Scheme 1). EIS characterization was performed using a frequency range of 0.1 Hz to 100 kHz with an applied AC potential amplitude of 0.1 V. The change in charge transfer resistance between the intermediate electrode configuration (BSA@anti-FGF-2@EDC/NHS@MWCNT-Au-Pt@cSPE) and the fully modified biosensor (FGF-2@BSA@anti-FGF-2@EDC/NHS@MWCNT-Au-Pt@cSPE) was monitored in the presence of 50 μL of a 5 mM equimolar [Fe(CN)₆]^3^⁻/^4^⁻ redox probe solution. The analytical objective was centered on detecting variations in interfacial electron-transfer resistance as an indicator of FGF-2 binding. All experimental procedures were conducted in triplicate, and the results were reported with standard deviation error bars to ensure reproducibility and reliability.

### Selectivity study

CEA and AFP of the cancer biomarkers are biological agents with the potential to interact with FGF-2. Therefore, these two agents were selected for interference studies and evaluated at a 1:1 ratio in the presence of 50 ng mL^−1^ FGF-2.

### Real sample application

A total of 30 saliva samples were collected among Muğla Sitki Koçman University students under standardized conditions, diluted 1:100, and used for analysis. The 1:100 dilution factor was preferred while preparing the real sample solutions in order to avoid the matrix effect as much as possible. To minimize variability, samples were obtained after oral cleansing and at least 2 h after any meal, following the chewing of pure sugar-free gum for 5–10 min to stimulate saliva flow. Each sample was spiked with FGF-2 at concentrations of 20, 50, and 75 ng mL^−1^, and all measurements were performed in triplicate. All experimental protocols were reviewed and approved by the Muğla Sıtkı Koçman University Medical and Health Sciences Ethics Committee (Protocol No. 240012; Decision No. 10). In order to reduce the overall cost of the developed biosensor and to evaluate the limits of the transducer, each electrode was used three times for each concentration. The requirement for informed consent was waived by the committee; however, informed consent was obtained from all participants and/or their legal guardians. All procedures were conducted in accordance with relevant institutional guidelines and regulations.

### Storage stability

The stability of the developed FGF-2 biosensors was evaluated using seven independent electrodes. For this purpose, the sensor response was monitored at defined time intervals following the immobilization of 50 ng mL^−1^ FGF-2 onto the electrode surface. Measurements were conducted daily over a period of 5 days and subsequently on a weekly basis for 1 month. Seven independently prepared immunosensors were stored at 4 °C and measured at predefined time points; between measurements, each sensor was rinsed with phosphate buffer and returned to storage.

### Statistical analysis

The normality of continuous variables was assessed using the Shapiro-Wilk test. Variables that did not conform to a normal distribution were summarized using median and range (minimum-maximum). Due to the non-normal distribution of the data, comparisons between two independent concentration groups were performed using the Mann-Whitney *U* test. All statistical analyses were conducted using a two-tailed approach, with a significance threshold set at *p* < 0.05. Data analysis was carried out using IBM SPSS Statistics software, version 27.0 (IBM Corp., Armonk, NY, USA).

## Results and discussion

### Characterization of MWNCT-Au-Pt hybrid nanoparticle

**Fig. 1:**
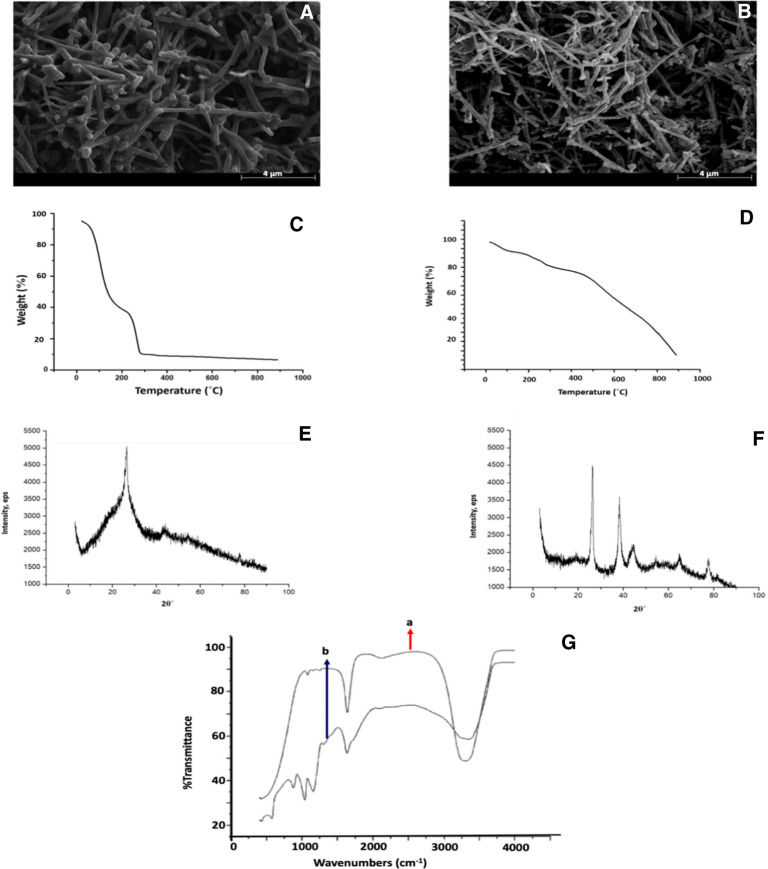
SEM images of activated MWCNT (**A**) and MWCNT-Au-Pt (**B**). TGA curves of MWCNT (**C**) and MWCNT-Au-Pt (**D**). XRD patterns of activated MWCNT (**E**) and MWCNT-Au-Pt (**F**). FTIR spectra (**G**) for activated MWCNT (a) and MWCNT-Au-Pt (b)

The surface morphologies of the activated MWCNTs and the MWCNT-Au-Pt hybrid nanomaterial were investigated using SEM (Fig. 1A and B). The SEM image of the activated MWCNTs (Fig. 1A) reveals a dense network of entangled nanotubes with clearly defined tubular structures. In contrast, the SEM image of the MWCNT-Au-Pt composite (Fig. 1B) shows that the nanotubes retain a semi-cylindrical morphology, with their surfaces uniformly decorated with agglomerated Pt and Au nanoparticles.

XRD patterns of the activated MWCNTs and MWCNT-Au-Pt nanocomposite are presented in Fig. 1E and F. The XRD pattern of the activated MWCNTs shows characteristic peaks that reflect their graphitic structure. In contrast, the MWCNT-Au-Pt nanohybrid exhibits additional diffraction peaks at approximately 38.18°, 44.36°, and 46.0°, which are consistent with the presence of Au nanoparticles [16, 17], while the peaks at 64.8° and 67.3° correspond to the characteristic crystallographic planes of Pt nanoparticles, as reported in previous studies [18]. These results confirm the successful deposition of Au and Pt nanoparticles onto the MWCNT structure.

TGA was utilized to investigate how the addition of metal nanoparticles influences the thermal stability of the activated MWCNTs, as shown in Fig. 1C and D. From Fig. 1C, it was obvious that weight losses were observed between 100 and 200 °C, and between 200 and 400 °C. The initial weight loss was attributed to the evaporation of water and the release of embedded solvents, whereas the second weight loss corresponded to the thermal decomposition of MWCNTs, which were fully carbonized at 350 °C under a nitrogen atmosphere. The data in Fig. 1D reveal that loading Au and Pt nanoparticles leads to a noticeable change in the degradation pattern of the MWCNTs, resulting in decreased thermal stability. Moreover, when the temperature was increased to a level higher, the crystal structure collapsed, resulting in a sharp drop in weight. This effect is likely due to the catalytic properties of the noble metals, which accelerate the oxidation and breakdown of the carbon nanotube structure, consistent with earlier reports in the literature [19, 20].

FTIR analysis (Fig. 1G) confirms the successful functionalization of the nanocomposites. The broad band around 3400 cm⁻^1^ indicates hydroxyl (–OH) groups introduced by acidic treatment, while the broad peak between 3400 and 3600 cm⁻^1^ suggests the presence of amine (–NH) groups. These findings are consistent with previous reports demonstrating the formation of hydroxyl and amine functionalities during oxidative treatment of carbon nanotubes. A strong absorption band near 1700 cm⁻^1^ confirms the presence of carboxylic acid (–COOH) groups, indicating successful oxygen-containing functionalization. Bands between 1000 and 1500 cm⁻^1^ correspond to C–O, C–N, and aromatic C=C vibrations, showing the presence of oxygen- and nitrogen-containing groups. In the MWCNT-Au-Pt sample, enhanced peaks in the 500–800 cm⁻^1^ region suggest metal-oxygen bonds, confirming the successful attachment of Au and Pt nanoparticles, in agreement with findings by previous studies [21–23].

### The surface characterization of FGF-2 biosensor

**Fig. 2:**
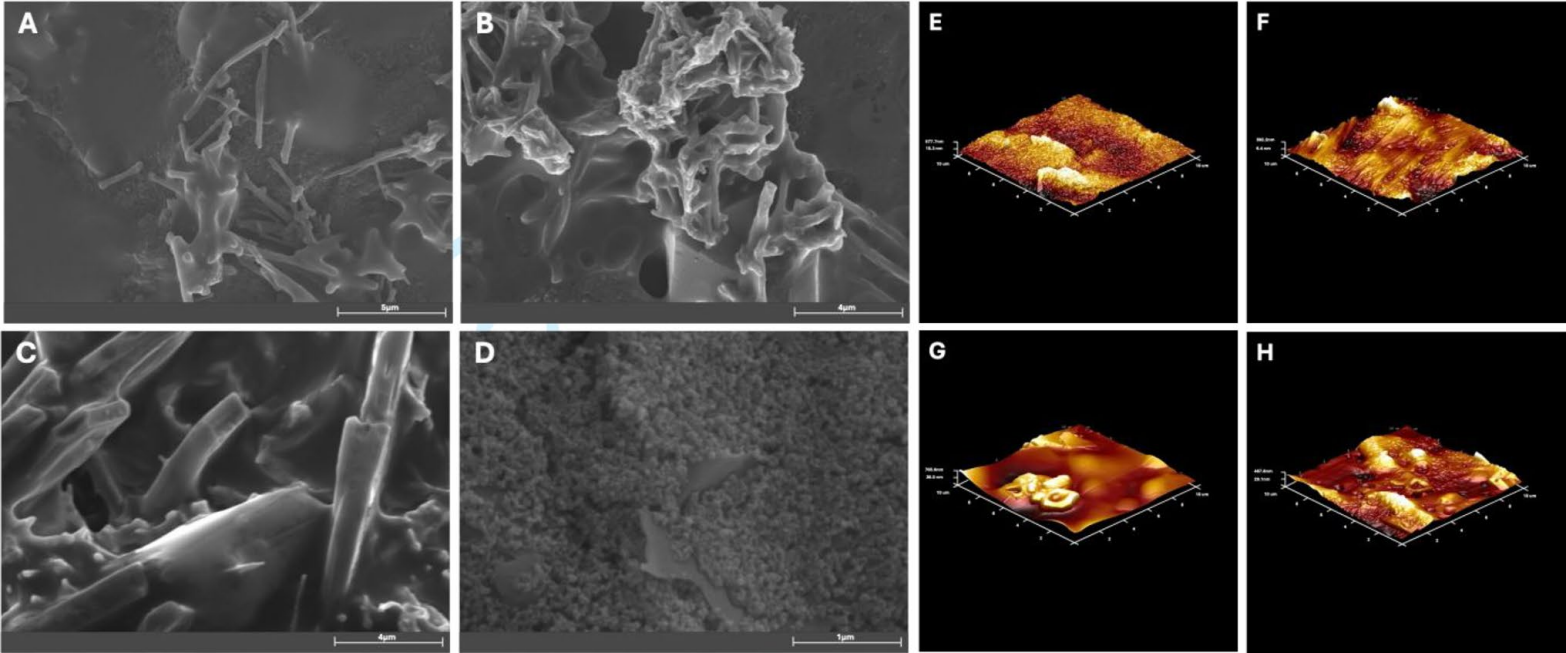
SEM images (**A**–**D**) and AFM images (**E**–**H**) of the electrode surfaces at each modification step: bare cSPE, MWCNT-Au-Pt@cSPE, anti-FGF-2@EDC/NHS@MWCNT-Au-Pt@cSPE, and FGF-2@BSA@anti-FGF-2@EDC/NHS@MWCNT-Au-Pt@cSPE

Figure 2A–D shows the SEM images and Fig. 2E–H shows the AFM images of cSPE at successive stages of surface modification as mentioned in the figure captions. As shown in Fig. 2A, the surface of the unmodified cSPE appears smooth and lacks distinguishable morphological features. In contrast, subsequent modification steps result in the appearance of spherical structures on the electrode surface, as observed in Fig. 2B–D, which are indicative of the successful immobilization of receptors and proteins. These stepwise modifications illustrate the structural changes on the electrode surface during the fabrication of the biosensor [24–28]. Throughout the various modification stages, Fig. 2E–H reveals the presence of small, grain-like particles and hill-like aggregations on the electrode surface. Notably, the AFM images shown in Fig. 2G and H, corresponding to the cSPE after anti-FGF-2 immobilization and subsequent FGF-2 binding, clearly indicate the formation of a distinct protein layer on the electrode surface [29, 30].

### Electrochemical characterization of the FGF-2 biosensor

EIS was utilized to characterize the electrochemical properties of the fabricated FGF-2 biosensor and to monitor each step of the surface modification process. The first step was to determine the most effective electrode structure. In order to do this, four types of FGF-2 immunosensors including MWCNT, MWCNT-Au, MWCNT-Pt, and MWCNT-Au-Pt were fabricated, and their stepwise responses against [Fe(CN)₆]^3^⁻/^4^⁻ redox probe were followed via EIS (Fig. 1S). Figure S1 presents the Nyquist plots illustrating the stepwise fabrication of the FGF-2 immunosensor using different MWCNT-based electrode platforms. As demonstrated in Figure S1, the modification of the cSPE surface with MWCNT, MWCNT-Au, and MWCNT-Pt results in a moderate charge transfer resistance difference between BSA addition and FGF-2 antigen addition, whereas MWCNT-Au-Pt dramatically decreases the charge transfer resistance compared to the other platforms and hence results in the highest resistance change in the end. The decrease in charge transfer resistance may be attributed to the enhanced electrical conductivity and synergistic catalytic effects provided by the MWCNT-Au-Pt nanocomposite, which facilitates electron transfer at the electrode interface, as previously reported [15, 31]. For this reason, MWCNT-Au-Pt was chosen as the hybrid nanomaterial for FGF-2 immunosensor fabrication (Fig. S1).

**Fig. 3:**
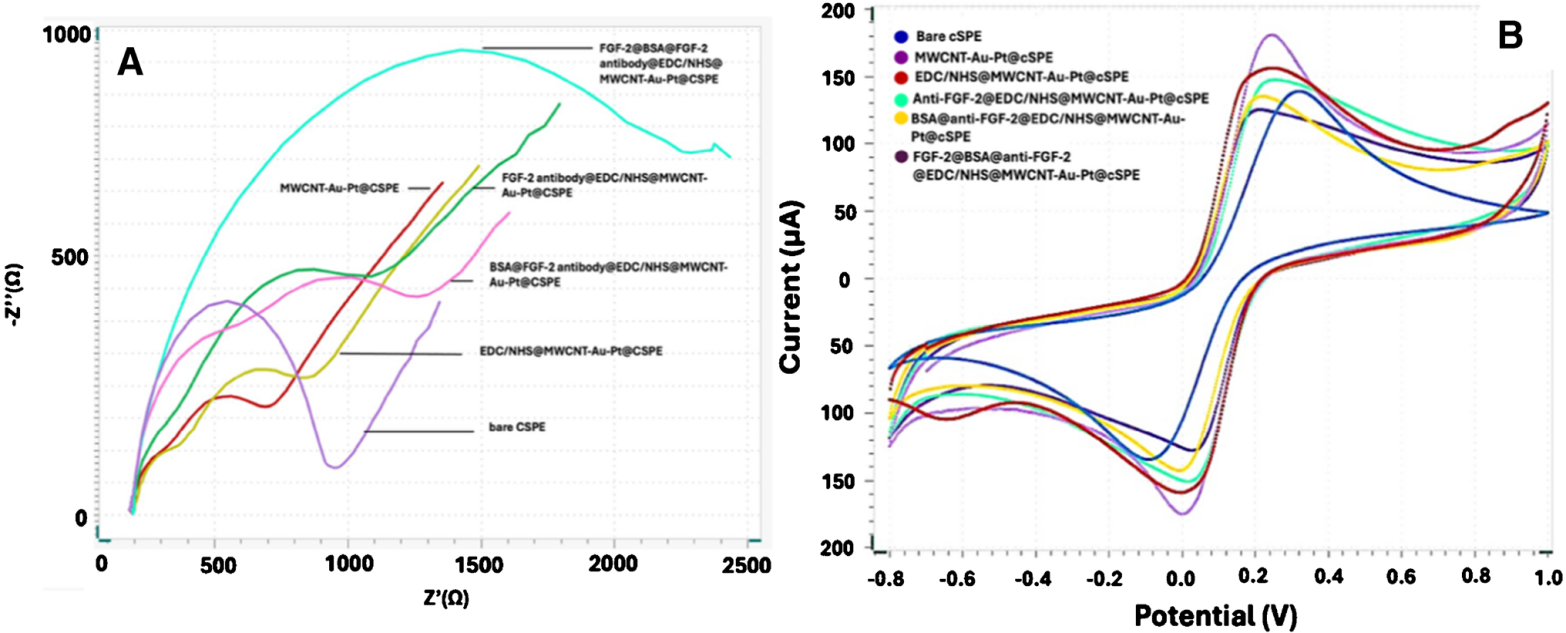
The electrochemical characterization of the impedimetric FGF-2 biosensor, including (**A**) the EIS and (**B**) the CV. CV measurements (0.0 V and 0.95 V) were conducted in 50 mM pH 7.4 phosphate buffer at 50 mV s^−1^ in the presence of 5 mM [Fe(CN)_6_]^3−/4−^ as a redox probe, and EIS measurements were performed in 50 mM pH 7.4 phosphate buffer including 50 μL 5 mM [Fe(CN)_6_]^3−/4−^ redox probe in the range of 0.1 Hz–10 kHz at 0.10 V potential. The FGF-2 immunosensor includes 6 μL of a 6 mg/mL MWCNT-Au-Pt; 10 μL of 100 mM EDC and 10 μL of 150 mM NHS; 10 μL of anti-FGF-2 0.2 μg/mL; 0.05 µg/mL of FGF-2; anti-FGF-2 incubation temperature 4 °C and incubation duration 60 min; anti-FGF-2-FGF-2 interaction time 30 min; incubation temperature 37 °C. For these experiments, all the electrodes were single use; each measurement was performed with a freshly prepared electrode

Moreover, Fig. 3A demonstrates the Nyquist diagram of more detailed MWCNT-Au-Pt-based FGF-2 immunosensor’s preparation steps. As can be seen from the figure, after the immobilization of MWCNT-Au-Pt onto the cSPE surface, the semi-circle domain decreases, and the MWCNT-Au-Pt@cSPE exhibited the smallest Nyquist semicircle, indicative of low charge transfer resistance (Rct) and high surface conductivity. Following activation with EDC/NHS, an increase in Rct was observed, suggesting a decrease in conductivity due to surface functionalization. Subsequent immobilization of anti-FGF-2 antibodies resulted in a further enlargement of the semicircle, reflecting increased interfacial resistance. The blocking of nonspecific binding sites using BSA led to two semicircles. In impedimetric biosensor systems, immobilization of a biomolecule on the electrode surface can hinder the interaction between the redox probe and the electrochemical double layer. This reduced electron-transfer efficiency typically appears as an increase in the R_CT_ value because the probe experiences more difficulty reaching the electrode interface. After BSA modification in our system, the appearance of two semicircles in the Nyquist plot may be attributed to two distinct charge transfer processes. The first, observed in the higher-frequency region, likely reflects the electron transfer occurring directly at the anti-FGF-2@MWCNT-Au-Pt modified electrode surface. The second semicircle in the lower-frequency region may correspond to electron transfer at the partially BSA-covered areas of the electrode, where the insulating protein layer further restricts charge movement [32]. Finally, incubation with FGF-2 caused a marked increase in Rct, consistent with the formation of the anti-FGF-2/FGF-2 complex and the consequent impediment to charge transfer. These stepwise increases in impedance collectively confirm the successful functionalization of the cSPE surface and the specific interaction between FGF-2 and its corresponding antibody.

Apart from EIS, CV was also employed to characterize the fabrication steps of the developed immunosensor in the presence of the [Fe(CN)₆]^3^⁻/^4^⁻ redox probe. As shown in Fig. 3B, the MWCNT-Au-Pt–modified electrode exhibits the highest current, indicating the most efficient electron-transfer kinetics. As expected, the sequential modification of the electrode surface results in a gradual decrease in current, confirming the successful formation of each layer, which progressively hinders electron transfer.

### Optimization studies

To ensure optimal performance and analytical accuracy, the electrochemical FGF-2 biosensor was systematically optimized by varying key parameters, including the amount of MWCNT-Au-Pt, the amount of anti-FGF-2 together with its incubation time, and the incubation conditions—both temperature and duration—for anti-FGF-2/FGF-2 antigen interactions. These optimization experiments were carried out in triplicate using EIS, employing 70 μL of a 5 mM [Fe(CN)₆]^3^⁻/^4^⁻ redox probe at a fixed potential of 0.1 V.

### MWCNT-Au-Pt amount

**Fig. 4:**
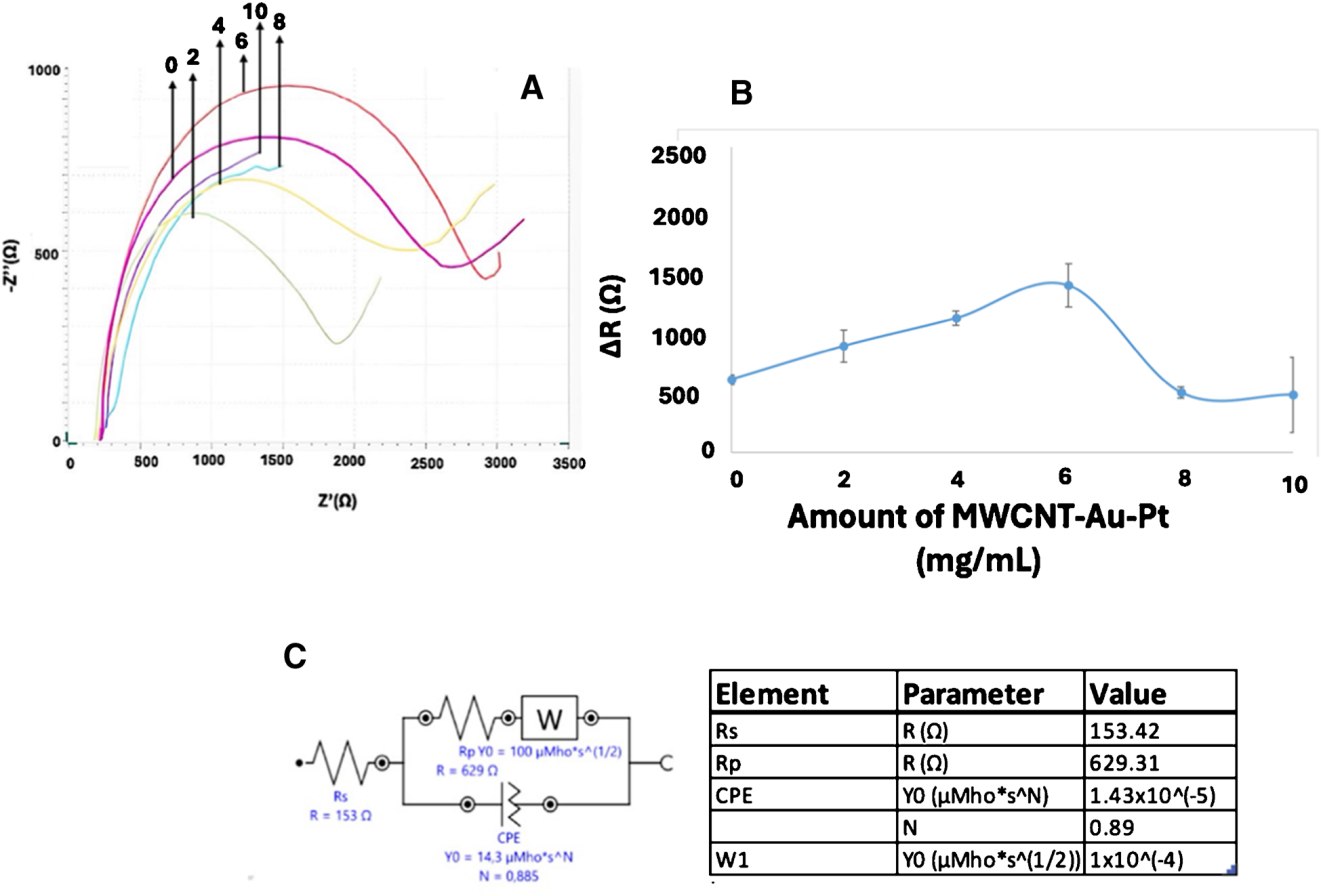
**A** EIS results for optimizing the MWCNT-Au-Pt amount (0, 2, 4, 6, 8, and 10 mg/mL). **B** The Excel plot of MWCNT-Au-Pt amount optimization. **C** The equivalent circuit model used for fitting, along with the corresponding parameter values and highlighting variations in charge transfer resistance (R_CT_) and diffusion behavior (Warburg element, W) (*n* = 3). Anti-FGF-2 0.5 μg/mL; all other conditions as in Fig. 3

The amount of MWCNT-Au-Pt hybrid nanomaterial integrated into the developed immunosensor was systematically optimized. To this end, six electrochemical FGF-2 immunosensors were fabricated, 6 µL MWCNT-Au-Pt hybrid nanomaterial suspension of each incorporating varying concentrations (0, 2, 4, 6, 8, and 10 mg/mL), along with a constant concentration of anti-FGF-2 (0.5 µg/mL). The electrochemical responses of these immunosensors were then evaluated against 0.05 µg/mL of FGF-2 (Fig. 4 and Figure S2). As illustrated in Fig. 4A and B, the immunosensor containing 6 mg/mL of the MWCNT-Au-Pt hybrid nanomaterial exhibited the highest response, and Figure S2 shows the Nyquist plots obtained for different concentrations of the MWCNT-Au-Pt hybrid nanomaterial (0, 2, 4, 6, 8, and 10 mg/mL), illustrating the difference between antigen and BSA signals. Notably, further increases in the amount of nanomaterial beyond 6 mg/mL led to a reduction in the resistance signal. This decline may be attributed to the oversaturation and non-uniform distribution of the nanomaterial on the electrode surface, potentially limiting the effective active surface area available for analyte interaction [33]. Meanwhile, Fig. 4C shows the term “diffusion behavior (Warburg element, W)” refers to the impedance component associated with the diffusion of electroactive species at the electrode-electrolyte interface.

### Anti-FGF-2 concentration

**Fig. 5:**
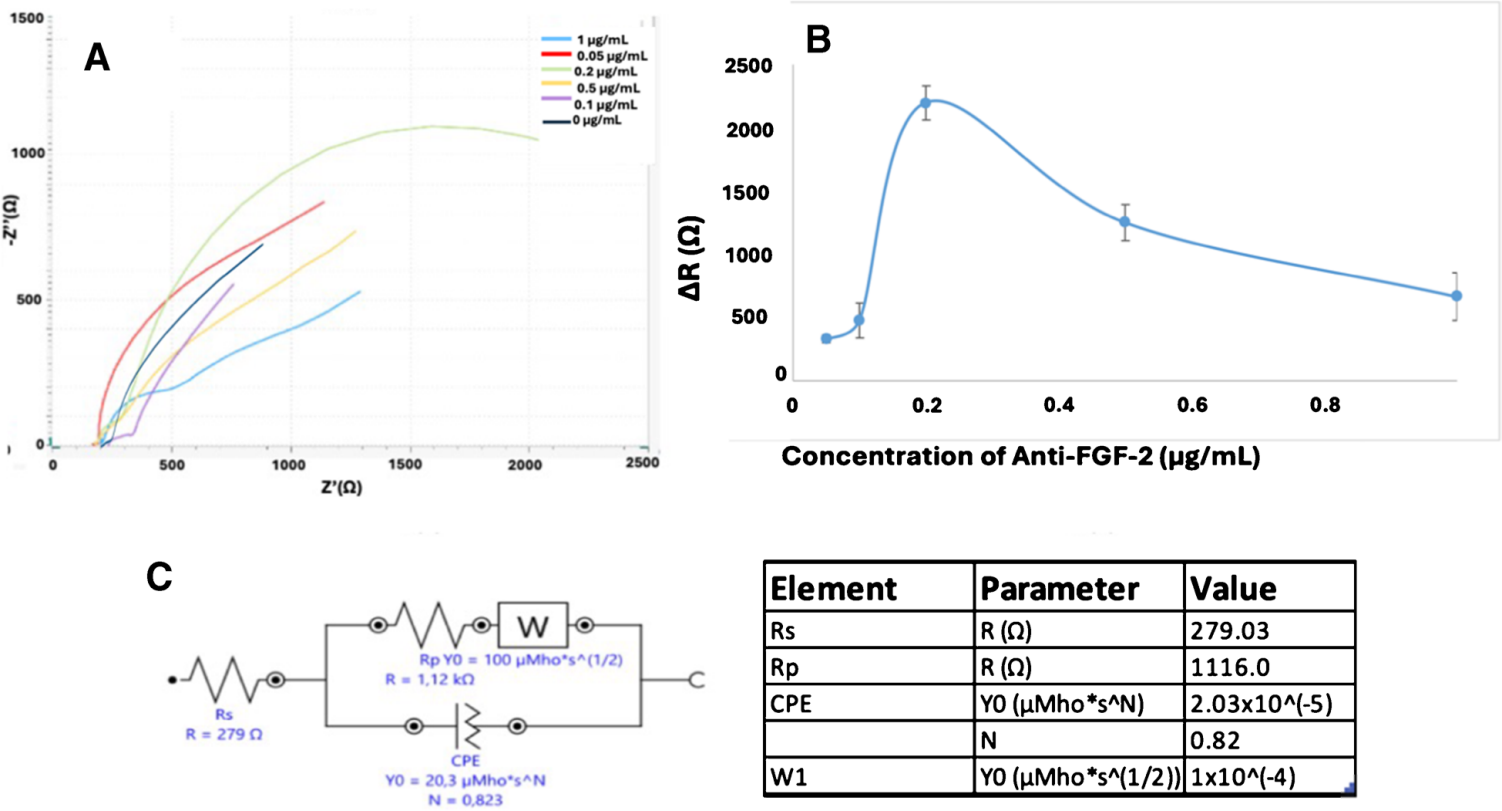
**A** Nyquist plots for different anti-FGF-2 concentrations (0, 0.05, 0.1, 0.2, 0.5, and 1 μg/mL), the corresponding Δ*R* variation indicating the optimal concentration (**B**), and the equivalent circuit model with fitted parameters used for EIS analysis (**C**) (*n* = 3). MWCNT-Au-Pt 6 mg/mL, all other conditions as in Fig. 3

To identify the optimal concentration of anti-FGF-2 for sensor fabrication, six electrodes were prepared with anti-FGF-2 at concentrations of 0, 0.05, 0.1, 0.2, 0.5, and 1 μg/mL. Among these, the electrode modified with 0.2 μg/mL anti-FGF-2 demonstrated the most prominent semicircle and the highest R_CT_, suggesting efficient antibody immobilization (Fig. 5 and Figure S3). Excessive accumulation of anti-FGF-2 on the electrode surface may lead to the obstruction of active binding sites, thereby hindering subsequent FGF-2 interaction. Over-saturation of the surface can impair antigen recognition, ultimately reducing the biosensor’s sensitivity and analytical reliability. To mitigate this effect, it is essential to optimize antibody loading and consider surface modification strategies that maintain the accessibility and functionality of binding sites [34]. Therefore, 0.2 μg/mL was selected as the optimal anti-FGF-2 concentration.

### Anti-FGF-2 incubation temperature

**Fig. 6:**
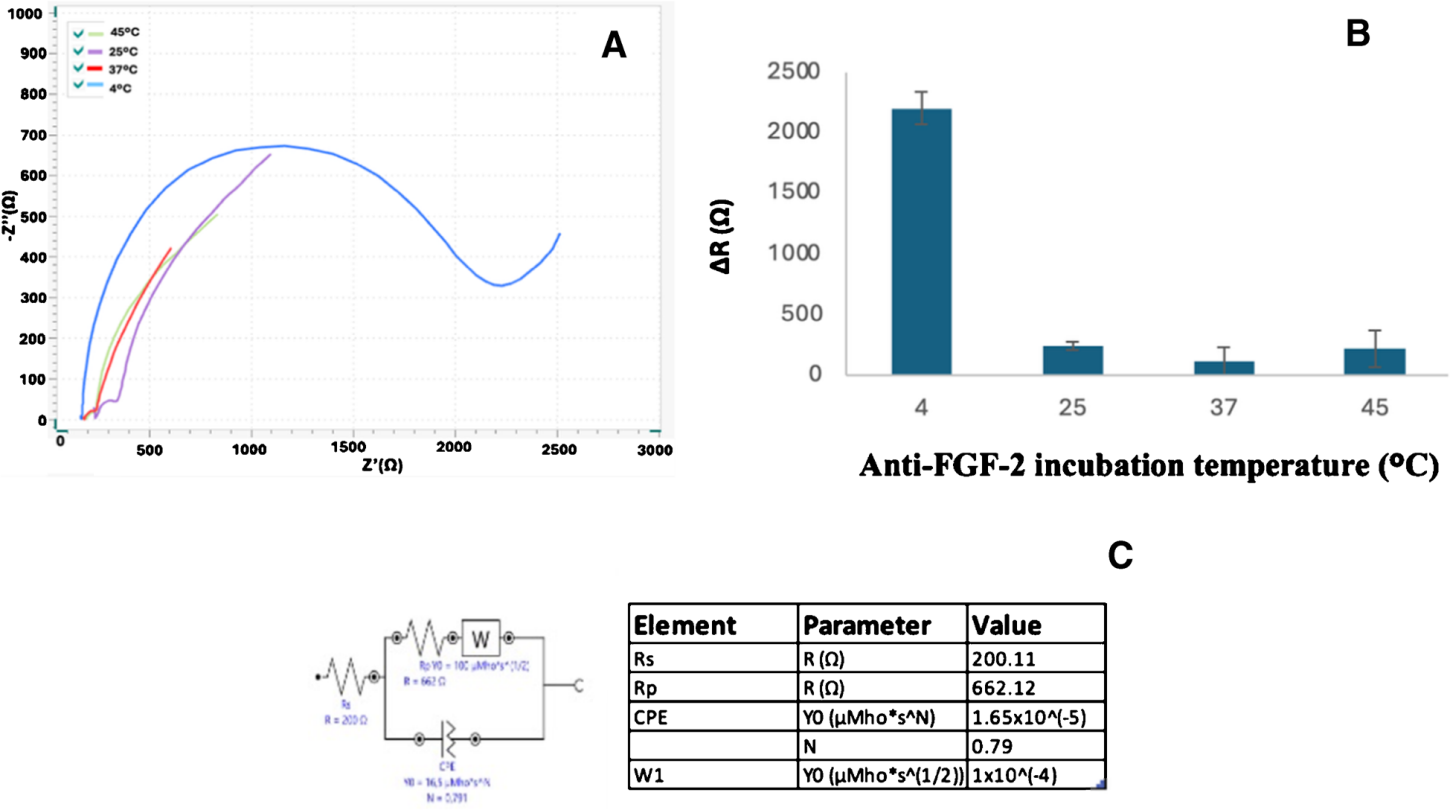
Nyquist diagrams of FGF-2 antibody incubation temperature were conducted at 4 °C, 25 °C, 37 °C, and 45 °C (**A**). The corresponding Δ*R* variation indicating the optimal temperature (**B**), and the equivalent circuit model with fitted parameters (**C**) (*n* = 3), all other conditions as in Fig. 3

The next parameter optimized was the immobilization temperature of anti-FGF-2 on the electrode surface. To evaluate this, anti-FGF-2 (0.2 μg/mL) was immobilized on the EDC/NHS-activated cSPE surface at 4 °C, 25 °C, 37 °C, and 45 °C for 60 min. As illustrated in Fig. 6, the highest impedance was observed at 4 °C, whereas higher temperatures resulted in lower resistance values. Therefore, 4 °C was selected as the optimal temperature for anti-FGF-2 immobilization (Fig. 6 and Figure S4).

### Anti-FGF-2 incubation duration

**Fig. 7:**
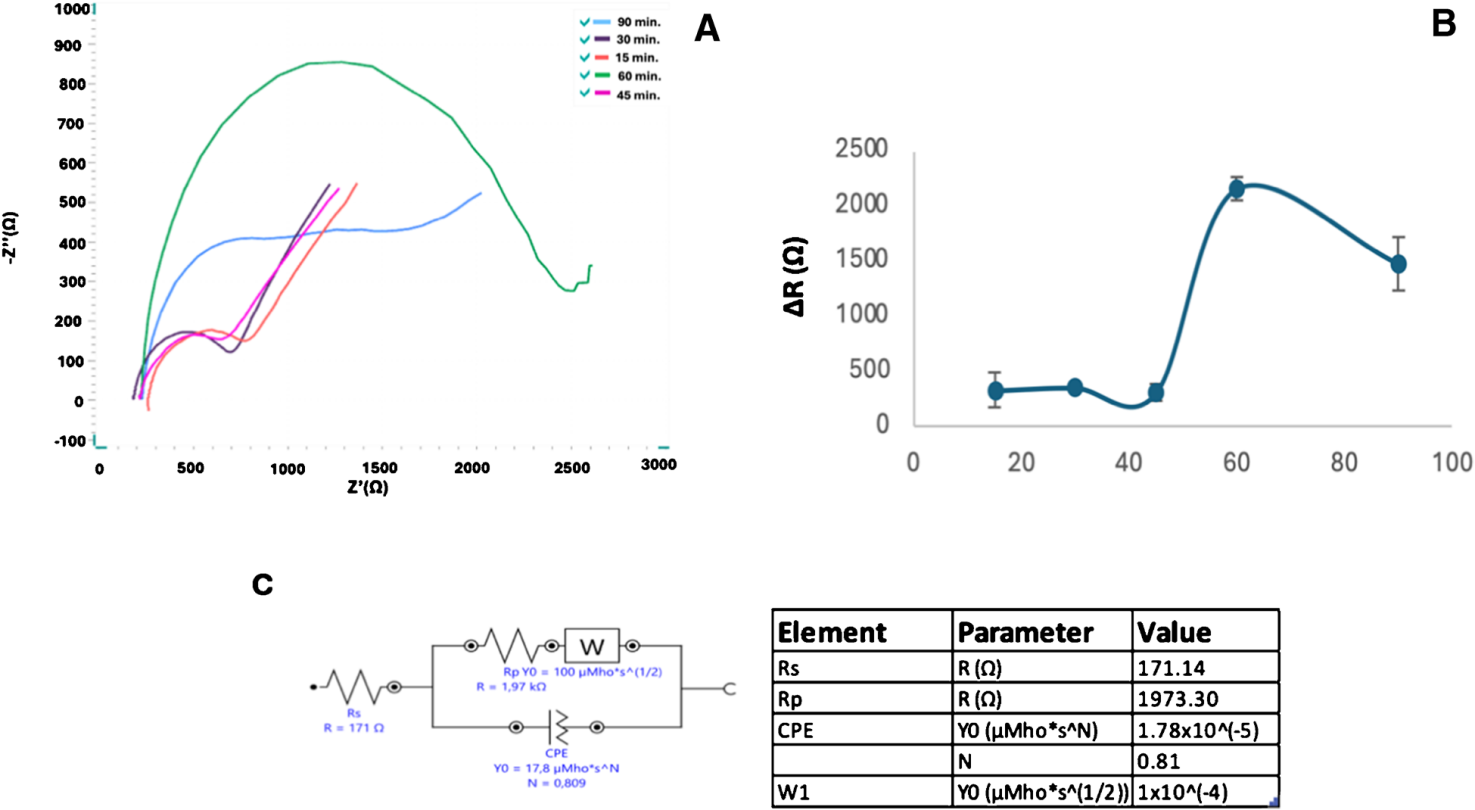
**A** Nyquist diagram for different incubation durations (15, 30, 45, 60, and 90 min.) and regarding resistance difference Excel plot (**B**) of anti-FGF-2 incubation time optimization studies for 15, 30, 45, 60, and 90 min. The equivalent circuit model with fitted parameters (**C**) (*n* = 3), all other conditions as in Fig. 3

As the second parameter for optimization, the incubation time of anti-FGF-2 was carefully evaluated. Five electrodes, prepared under previously established optimal conditions, were incubated with 10 μL of 0.2 μg/mL anti-FGF-2 solution (in 100 mM phosphate buffer, pH 7.4) for varying durations of 15, 30, 45, 60, and 90 min at 4 °C. The results revealed a progressive increase in resistance values up to 60 min, followed by a decline at longer incubation times (Fig. 7). This decrease is likely due to the gradual aggregation of bound anti-FGF-2 molecules on the electrode surface, which may ultimately cause their detachment [34]. Therefore, 60 min was selected as the optimal incubation time for subsequent experiments (Fig. 7 and Figure S5).

### FGF-2 incubation temperature

**Fig. 8:**
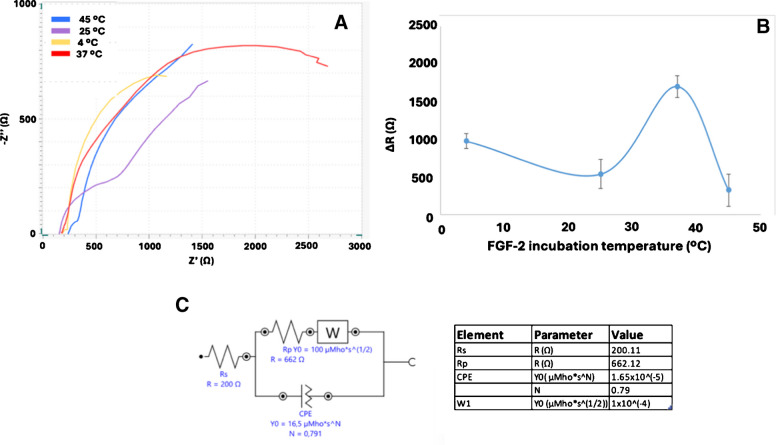
Nyquist diagrams obtained at different FGF-2 incubation temperatures (4 °C, 25 °C, 37 °C, and 45 °C) (**A**); the corresponding Δ*R* variation identifying the optimal temperature (**B**); the system was fitted using an equivalent circuit model (**C**) (*n* = 3). All other conditions as in Fig. 3

The efficiency of the interaction between anti-FGF-2 and FGF-2 is strongly influenced by critical factors such as incubation temperature (4, 25, 37, 45 °C), which can notably affect the biosensor’s sensitivity. As shown in Fig. 8 and Figure S6, the immunosensor exhibited the highest impedance response when incubated with FGF-2 at 37 °C. This indicates that 37 °C represents the optimal temperature for the anti-FGF-2/FGF-2 interaction on the electrode surface, most likely due to enhanced molecular interactions and favorable reaction kinetics at this condition [35].

### FGF-2 incubation time

**Fig. 9:**
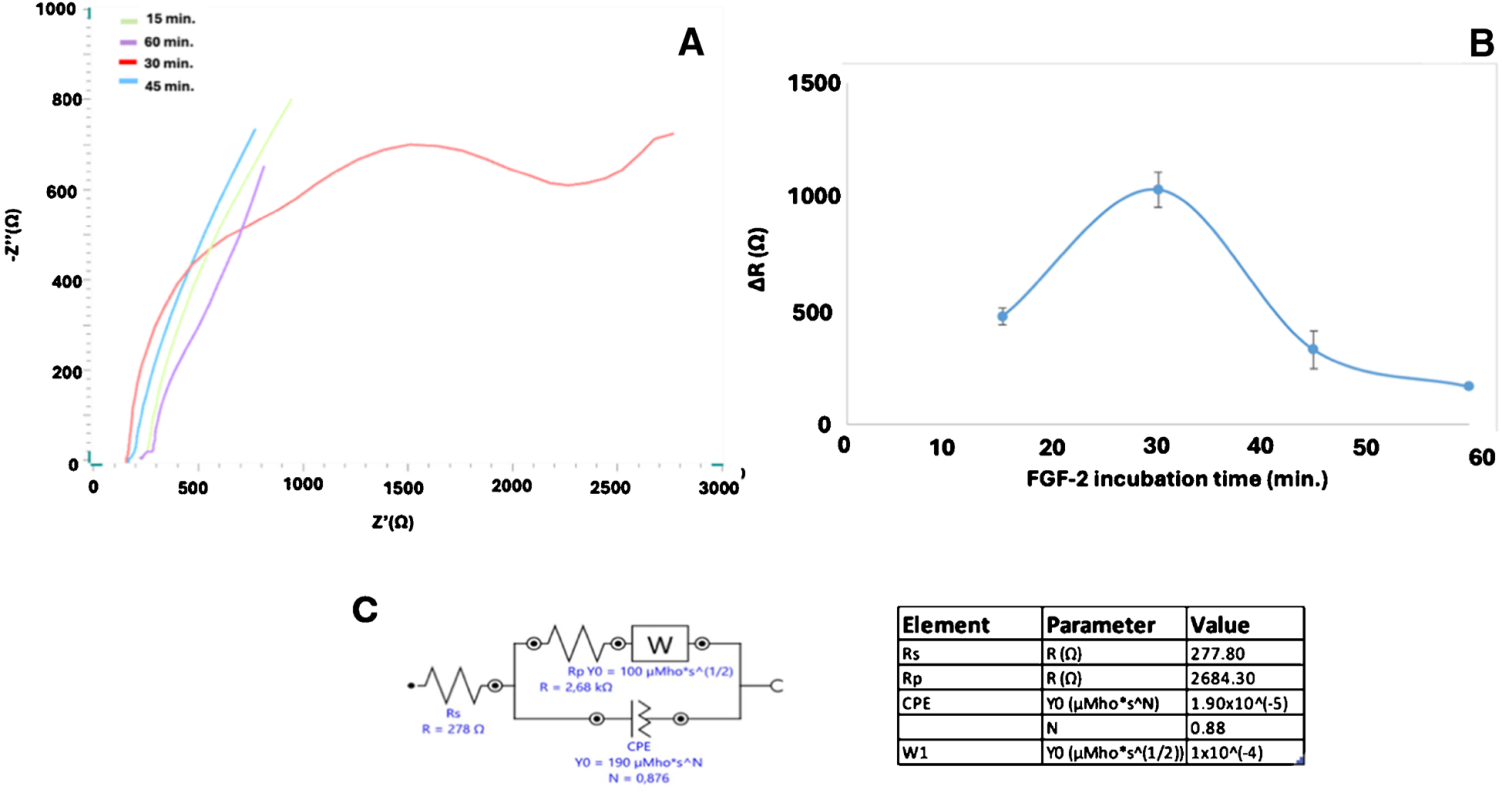
Nyquist diagram (**A**) and impedance difference Excel plot (**B**) of FGF-2 incubation temperature optimization studies for 15, 30, 45, and 60 min. **C** At the optimal 30-min incubation, the system is modeled with an equivalent circuit (*n* = 3); all other conditions are as in Fig. 3

Since correct measurement of bioactive layer concentration (FGF-2) in the developed biosensor system is the main aim of this constructed immunosensor, optimizing the incubation time is also essential. To assess this, the biosensor prepared under optimal conditions was incubated with 0.05 μg/mL FGF-2 (in pH 7.4 phosphate buffer) for 15, 30, 45, and 60 min at 37 °C. Resistance measurements indicated that the most effective electrode coverage occurred at 30 min. However, incubation beyond 30 min resulted in a decrease in resistance, possibly due to the gradual detachment of aggregated FGF-2 molecules from the electrode surface over time (Fig. 9 and Figure S7). Therefore, 30 min was selected as the optimal incubation time for subsequent experiments.

### Analytical characteristics

**Fig. 10:**
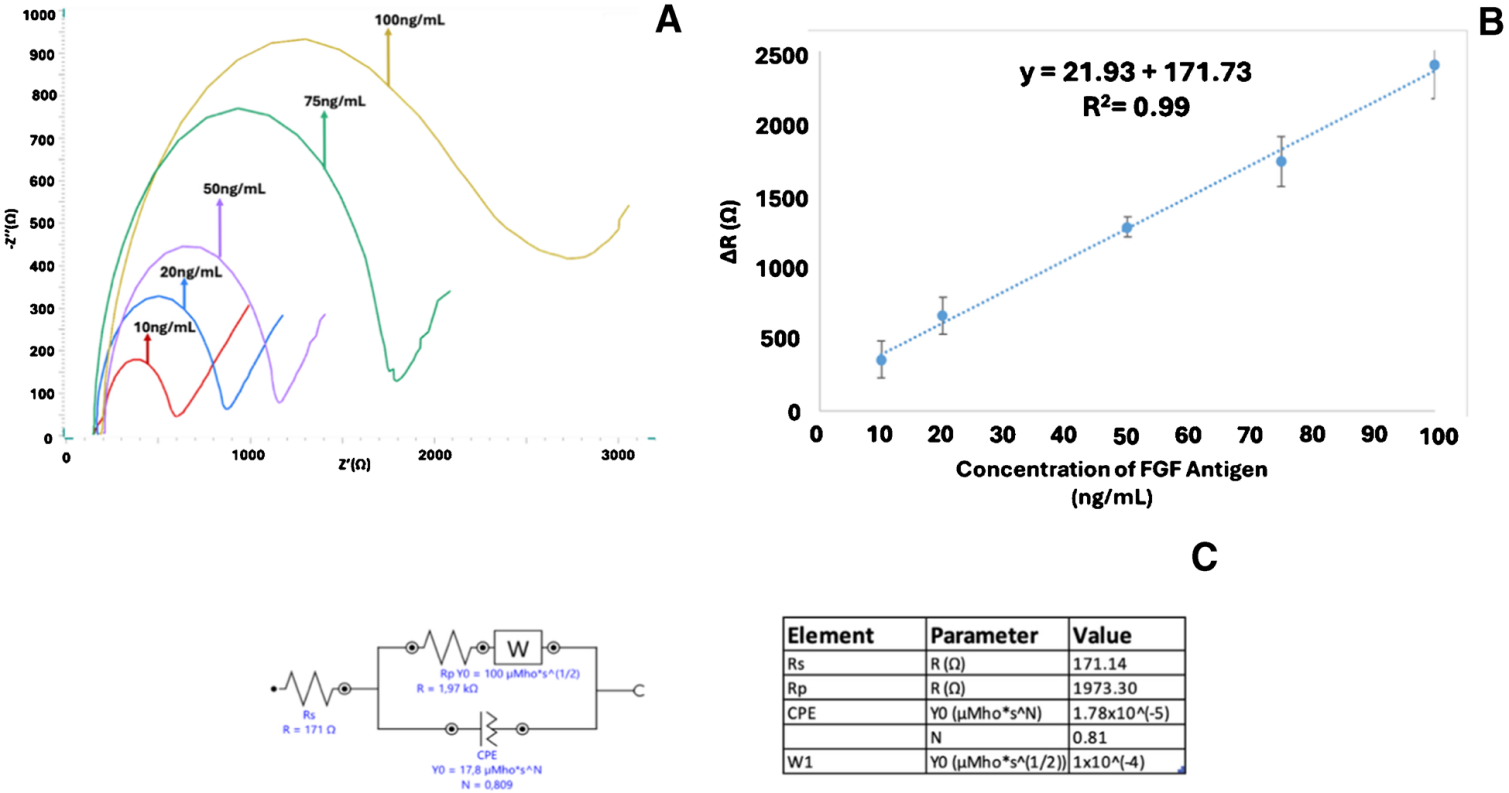
**A**1–**A**6 Nyquist diagram and (**B**) calibration curve corresponding to FGF-2 concentrations of 10, 20, 50, 75, and 100 ng mL^−1^. **C** Equivalent circuit model used to fit the experimental data (*n* = 3). All experimental conditions were applied as specified in Fig. 3

After optimizing the experimental conditions, the linear detection range of the FGF-2 biosensor was assessed using FGF-2 protein solutions at concentrations ranging from 10 to 100 ng/mL (Fig. 10 and Figure S8). As shown in Fig. 10, the biosensor demonstrated a linear response over this range, characterized by the calibration equation *y* = 21.93*x* + 171.73 with a correlation coefficient of *R*^2^ = 0.99.

To evaluate the sensitivity of the biosensor, the limit of detection (LOD) and limit of quantification (LOQ) were determined. Using the formulas LOD = 3 s/m and LOQ = 10 s/m, where s is the standard deviation of the blank and m is the slope of the calibration curve, the LOD and LOQ were calculated as 1.01 ng mL⁻^1^ and 3.10 ng mL⁻^1^, respectively.

In addition, the relative standard deviation (RSD) for an FGF-2 protein concentration of 50 ng/mL was determined to be 5.10% (*n* = 3), indicating reliable reproducibility. The analytical performance of the developed FGF-2 biosensor was compared with those reported in previous studies (Table 1). Although surface plasmon resonance imaging (SPRi) detection methods generally achieve lower detection limits, the biosensor developed in this work offers notable advantages in terms of simplicity, speed, and practical applicability.

**Table 1:** Comparison of analytical performance of the developed FGF-2 immunosensor with other sensors for FGF-2 detection

Biosensor	Transduction element	Sensing strategy	Method	Linear range	Detection limit	References
Surface plasmon resonance imaging (SPRi) detection	Biosensor	Label-free immunosensor	-	5–80 pg/mL ng/mL	1.64 pg/mL	[12]
Microfluid chip	Electrochemical	Label-free immunosensor	Frequency	1–25 µg/mL	130 ng/mL	[36]
cSPE/MWCNT-Au-Pt/anti-FGF-2/BSA/FGF-2	Electrochemical	Immunosensor	EIS	10–100 ng/mL	4.81 ng/mL	This study

### Interference studies

Interference studies were carried out under optimized experimental conditions using 0.05 μg/mL FGF-2. Potential interfering substances—AFP and CEA—were selected based on literature and individually tested at concentration ratios of 1:1 relative to FGF-2. EIS measurements with these mixtures yielded recovery values of 98.55% and 96.54% for AFP and CEA, respectively, demonstrating minimal interference and high specificity of the biosensor. Three replicates were performed throughout all interference studies.

### Real sample application

To evaluate the performance of the developed biosensor in real sample analysis, FGF-2 was spiked into 30 human diluted saliva samples at concentrations of 20, 50, and 75 ng/mL. The results are summarized in Table 2. Since the same electrode was used with intermediate washings, the standard deviation values were found to be high in some measurements. Nevertheless, RSD values were consistently below ± 10%, indicating high reproducibility and reliability. These findings confirm the effectiveness of the biosensor for accurate determination of FGF-2 in real human diluted saliva samples.

**Table 2:** FGF-2 spiked real diluted saliva samples and their concentrations, resistance, RSD (%), and recovery (*R* (%)) values

Sample	Concentration (ng/mL)	Results (ΔΩ)	RSD (%) (*n* = 3)	*R* (%)
1	20	698 ± 55	7.90	105.32
	50	1473 ± 110	7.52	94.87
	75	1800 ± 83	4.65	104.53
2	20 50 75	699 ± 52 1474 ± 108 1799 ± 83	7.47 7.38 4.62	105.45 115.14 103.63
3	20	767 ± 7	0.94	96.39
	50	1509 ± 122	8.11	117.90
	75	1650 ± 74	4.52	95.02
4	20	867 ± 7	0.83	130.88
	50	1609 ± 122	7.61	125.70
	75	1783 ± 112	6.31	102.69
5	20	967 ± 7	0.74	145.96
	50	1443 ± 109	7.57	112.69
	75	1783 ± 0.97	0.98	102.69
6	20	731 ± 61	8.35	110.34
	50	1573 ± 110	7.04	122.88
	75	1900 ± 83	4.40	109.44
7	20	765 ± 7	0.94	115.51
	50	1509 ± 116	7.70	117.89
	75	1755 ± 52	3.01	101.07
8	20	887 ± 59	6.70	93.87
	50	1577 ± 10	0.66	123.21
	75	1734 ± 24	1.41	99.89
9	20	793 ± 33	4.27	119.71
	50	1600 ± 32	2.05	124.96
	75	1743 ± 39	2.25	100.38
10	20	870 ± 108	12.51	130.30
	50	1662 ± 39	2.38	129.82
	75	1721 ± 26	1.55	99.11
11	20	868 ± 42	4.89	130.95
	50	1606 ± 69	4.33	125.46
	75	1772 ± 23	1.31	102.05
12	20	855 ± 22	2.62	129.02
	50	1628 ± 48	2.99	127.16
	75	1745 ± 41	2.36	100.56
13	20	830 ± 51	6.15	105.24
	50	1619 ± 19	1.19	116.47
	75	1748 ± 30	1.75	100.66
14	20	897 ± 63	7.04	105.45
	50	1628 ± 32	2.03	97.14
	75	1746 ± 31	1.79	100.55
15	20	910 ± 29	3.25	97.33
	50	1590 ± 10	0.65	104.90
	75	1798 ± 0.99	0.55	103.58
16	20	929 ± 10	1.11	99.23
	50	1656 ± 16	1.02	109.56
	75	1752 ± 20	1.16	100.91
17	20	780 ± 17	2.23	104.07
	50	1630 ± 35	2.16	98.92
	75	1727 ± 13	0.79	99.46
18	20	933 ± 45	4.92	112.73
	50	1612 ± 15	0.94	98.95
	75	1748 ± 11	1.83	100.68
19	20	884 ± 13	7.19	100.67
	50	1639 ± 39	3.04	108.92
	75	1741 ± 39	2.28	100.28
20	20	852 ± 48	5.74	128.56
	50	1645 ± 42	2.56	128.45
	75	1748 ± 34	1.95	100.68
21	20	826 ± 63	7.69	92.67
	50	1625 ± 39	2.44	96.34
	75	1773 ± 19	1.66	102.11
22	20	826 ± 4	5.08	113.91
	50	1644 ± 23	1.77	117.67
	75	1749 ± 30	1.73	100.76
23	20	935 ± 6	1.29	102.58
	50	1635 ± 12	1.46	111.34
	75	1730 ± 5	0.29	99.29
24	20	901 ± 10	7.25	107.89
	50	1610 ± 26	1.65	100.74
	75	1753 ± 32	1.84	100.95
25	20	872 ± 102	11.79	131.54
	50	1607 ± 59	3.66	125.50
	75	1751 ± 52	2.99	100.88
26	20	901 ± 14	9.89	93.78
	50	1532 ± 39	9.63	99.01
	75	1795 ± 61	3.77	103.39
27	20	873 ± 34	3.91	114.73
	50	1573 ± 9	7.45	122.88
	75	1831 ± 70	3.81	105.45
28	20	853 ± 34	3.98	113.69
	50	1553 ± 34	6.99	121.32
	75	1711 ± 70	0.84	104.28
29	20	782 ± 7	0.84	100.12
	50	1489 ± 86	6.01	99.16
	75	1697 ± 18	1.09	97.72
30	20	846 ± 35	4.13	127.62
	50	1527 ± 92	6.01	119.25
	75	1682 ± 18	1.06	96.89

When comparing the 20 and 75 concentration groups, FGF-2 levels were found to be nearly doubled in the high-concentration group (median 861.463 (698.2–967.7) vs. 1749.24 (1650.2–1900.6), *p* < 0.001). In addition, root mean square error (RMSE) values were significantly higher in the low-concentration group and markedly lower in the high-concentration group (198.54 (35.25–304.73) vs. 17.57 (1.95–163.95), *p* < 0.001). These findings indicate that higher concentration not only increases FGF-2 levels but also reduces measurement error.

### Stability

For the stability study, the developed sensor was evaluated over a 1-month period using 50 ng mL⁻^1^ FGF-2. A total of seven independently prepared sensors (all modified with anti-FGF-2) were stored at 4 °C until use. The first three sensors were analyzed on days 1, 2, and 3, respectively. The remaining sensors were measured at the end of weeks 1, 2, 3, and 4. At each time point, measurements were performed in triplicate (*n* = 3), where the replicates correspond to three repeated readings obtained from the same sensor. The recovery values are presented in Table 3. The highly acceptable recovery values observed throughout the experiments indicate that the developed sensor can be reliably used as a stable and reproducible analytical platform over extended storage periods.

**Table 3:** Stability analysis of the developed sensor over a 1-month period

Time point	*R* (%)
Day 1	105.14
Day 2	91.52
Day 3	94.38
Week 1	100.14
Week 2	102.43
Week 3	102.73
Week 4	103.95

## Conclusion

The precise and accurate detection of anxiety disorder is still challenging. For this reason, the identification and the usage of suitable biomarkers for diagnosis is important. In this work, a MWCNT-Au-Pt-based impedimetric FGF-2 immunosensor was developed, optimized, and applied for FGF-2 detection in real diluted human saliva samples. Here, the real human saliva samples were analyzed using appropriate dilution to minimize matrix interference, and within the experimentally spiked concentration window, the developed immunosensor biosensor demonstrated practical applicability, accurate recovery, and reliable signal behavior. Considering the analytical characteristic parameters, hence the recovery values that were obtained from 30 diluted real human saliva samples, it can be concluded that a robust and effective biosensor that has diagnostic tool potential was developed for anxiety disorder determination. Since disposable cSPE was the backbone of this biosensor and it is facile to adapt cSPE to a portable potentiostat, it has the potential to become a point-of-care diagnostic device. Unlike conventional questionnaire-based assessments, this approach enables accurate and personalized diagnosis of anxiety disorders using only a single drop of properly diluted saliva.

## Supplementary Information

Below is the link to the electronic supplementary material.

ESM 1 Supplementary Material 1(DOCX 3.04 MB)

## Data Availability

The datasets used and/or analyzed during the current study are available from the corresponding author on reasonable request.

## Source of biological material

Human saliva.
