# Supplementary material for: MWCNT-Au-Pt hybrid nanocomposite–based electrochemical immunosensor for FGF-2 detection: a novel strategy for anxiety disorder diagnosis
Source: Anal Bioanal Chem. 2026 Feb 9;418(8):2231–46. doi: 10.1007/s00216-026-06346-z (PMC13065547; doi:10.1007/s00216-026-06346-z)
Supplement: Supplementary file 1 — Supplementary Material 1(DOCX 3.04 MB) [file 216_2026_6346_MOESM1_ESM.docx]

**SUPPORTING INFORMATION**

**MWCNT–Au–Pt Hybrid Nanocomposite-Based Electrochemical Immunosensor for FGF-2 Detection: A Novel Strategy for Anxiety Disorder Diagnosis**

Nil Su ÇAYLAYIK^1^ , Vasfiye Hazal ÖZYURT ^2,3^ , Burak Ekrem ÇİTİL ^4^, Ülkü ANIK^1,2*^

^1^ Faculty of Science, Chemistry Department, Mugla Sitki Kocman University, 48000 Kotekli, Mugla, Türkiye

^2^ Research Laboratory Center, Mugla Sitki Kocman University, Sensors, Biosensors and Nano-Diagnostic Systems Laboratory, Kotekli-Mugla, Türkiye

^3^ Faculty of Tourism, Department of Gastronomy and Culinary Arts, Mugla Sitki Kocman University, Kotekli, Mugla, Türkiye

^4^ Faculty of Medicine, Department of Medical Microbiology, Mugla Sitki Kocman University, Kotekli, Mugla, Türkiye

Corresponding author: Ülkü ANIK

* Correspondence to Faculty of Science, Chemistry Department, Mugla Sitki Kocman University, 48000 Kotekli, Mugla, Türkiye and Research Laboratory Center, Mugla Sitki Kocman University, Sensors, Biosensors and Nano-Diagnostic Systems Laboratory, Kotekli-Mugla, Türkiye

*E-mail addresses: ulkuanik@mu.edu.tr*

**
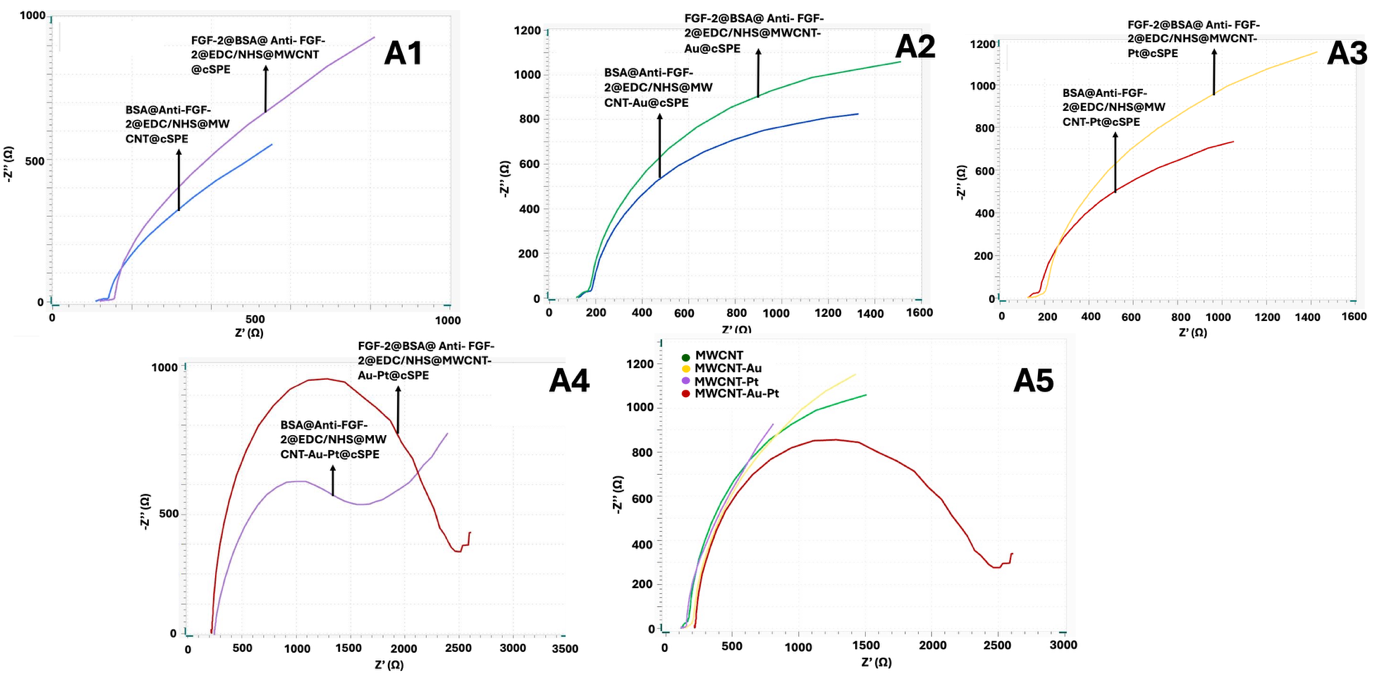
**

**Figure S1.** Nyquist plots showing the stepwise fabrication of FGF-2 immunosensors using different MWCNT-based electrode platforms. A1–A4 present the impedance responses recorded in the absence and presence of the analyte for MWCNT, MWCNT-Au, MWCNT-Pt, and MWCNT-Au-Pt modified cSPEs, respectively. A5 compares the final sensor responses of all electrode types, highlighting the effect of nanocomposite composition on charge-transfer resistance.


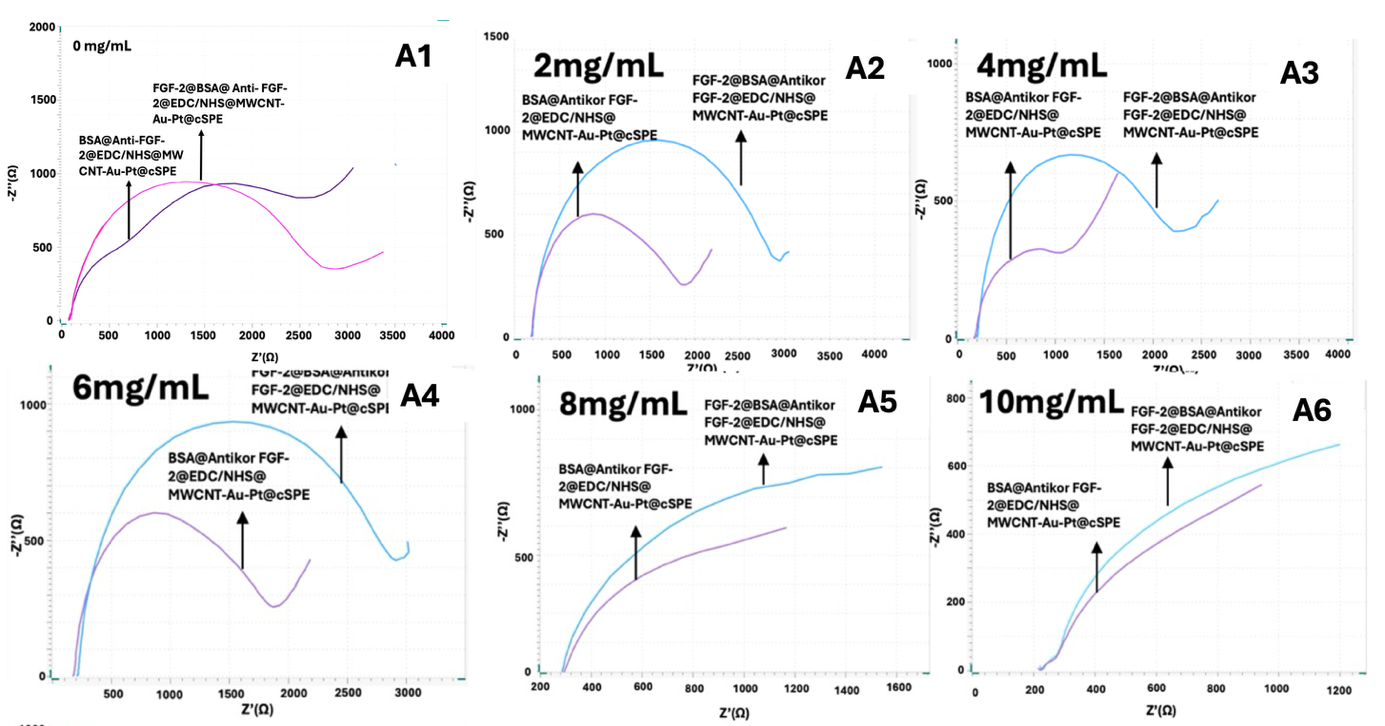


**Figure S2.**Nyquist plots obtained for different MWCNT–Au–Pt nanocomposite concentrations—0 mg/mL (A1), 2 mg/mL (A2), 4 mg/mL (A3), 6 mg/mL (A4), 8 mg/mL (A5), and 10 mg/mL (A6)—showing the impedance responses.


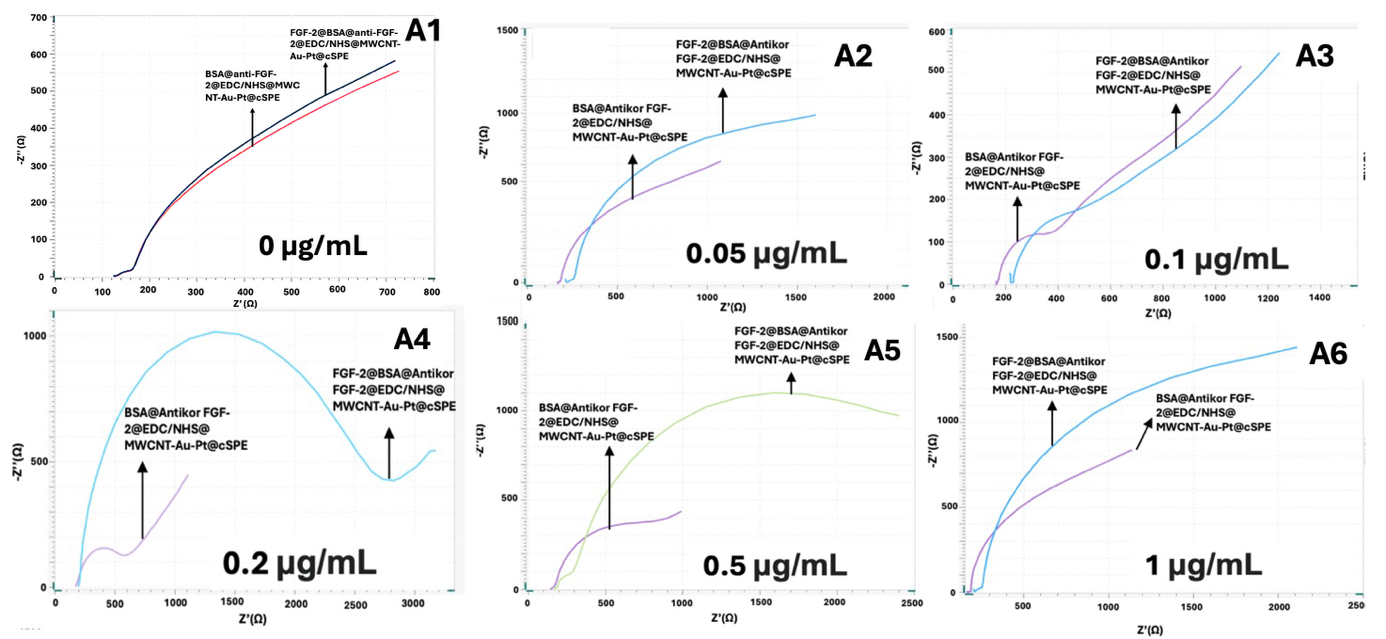


**Figure S3.** Nyquist plots recorded at different anti-FGF-2 concentrations—0 µg/mL (A1), 0.05 µg/mL (A2), 0.1 µg/mL (A3), 0.2 µg/mL (A4), 0.5 µg/mL (A5), and 1 µg/mL (A6)—illustrating the impedance changes.


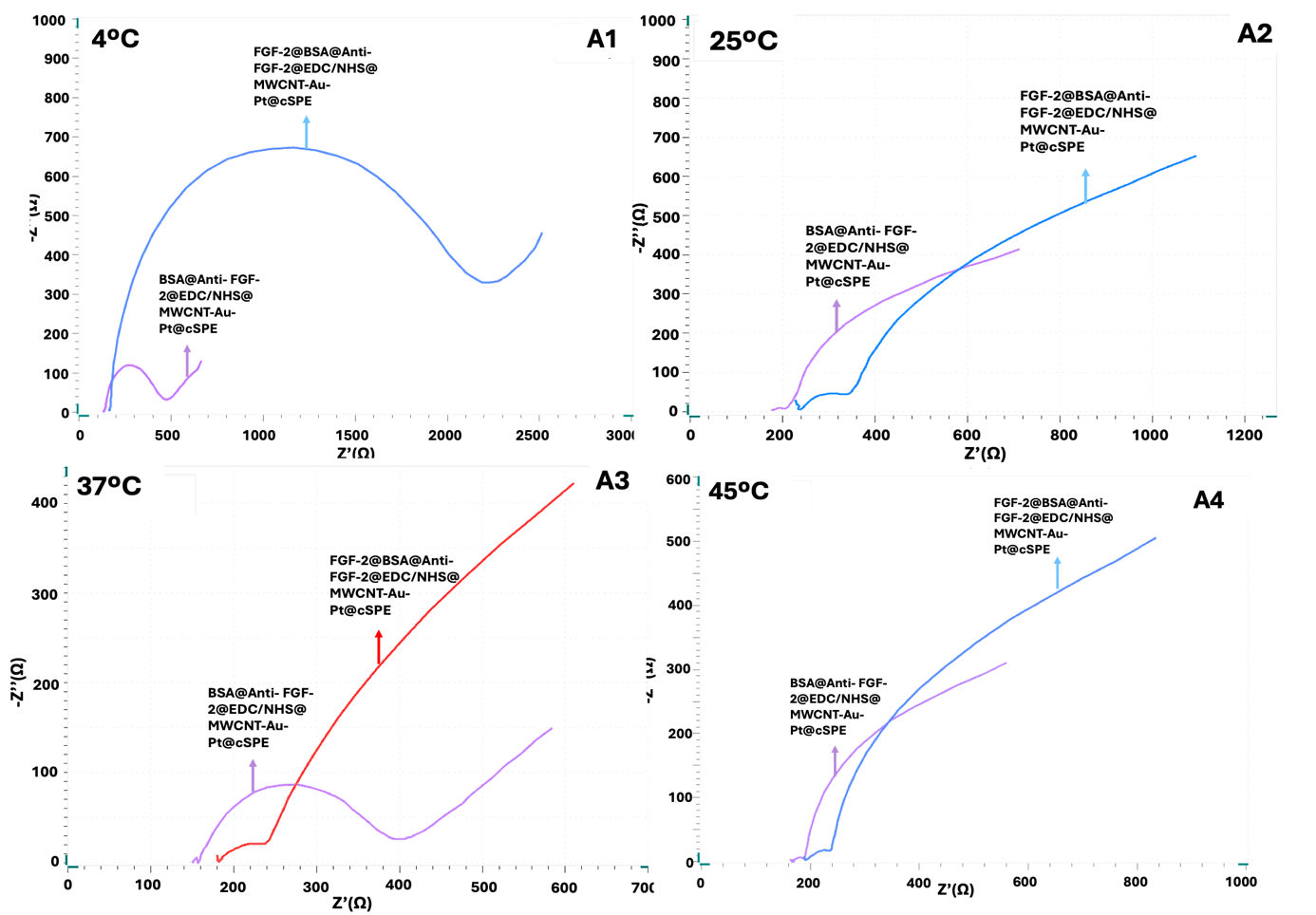


**Figure S4.** Nyquist plots recorded at different anti-FGF-2 incubation temperatures—4 °C (A1), 25 °C (A2), 37 °C (A3), and 45 °C (A4)—showing the impedance responses.

**
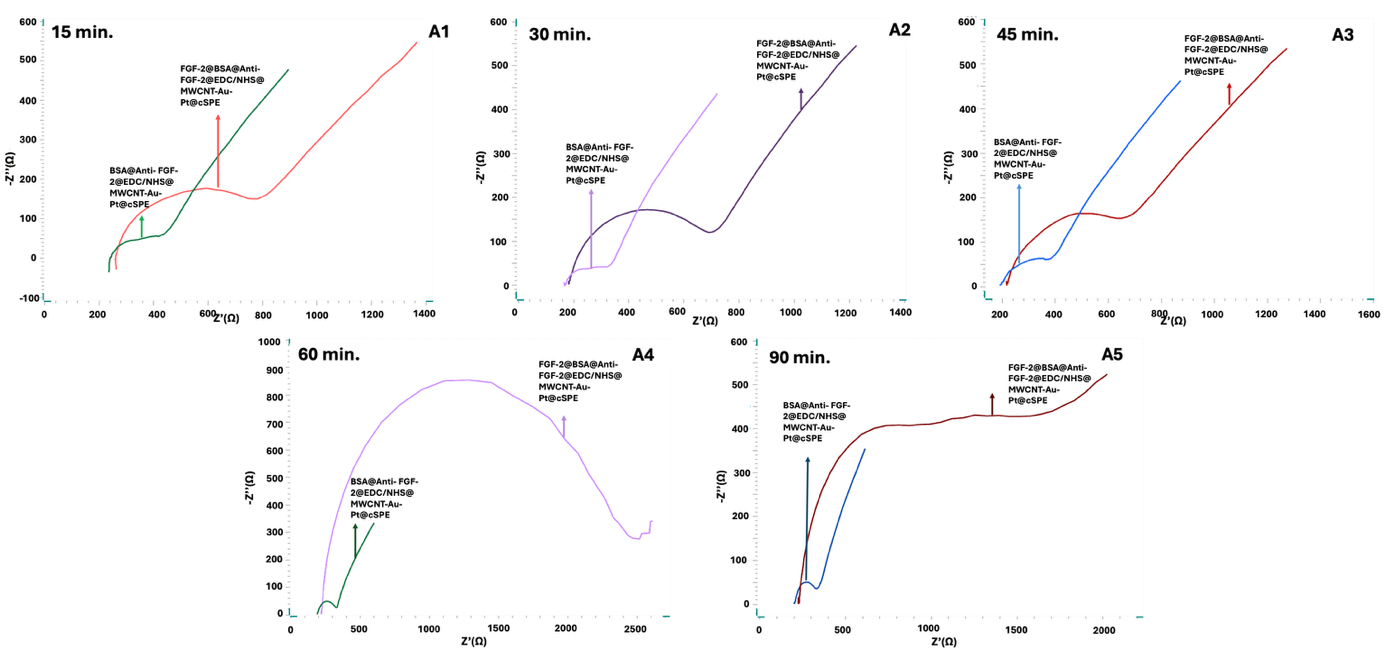
**

**Figure S5.** Nyquist plots recorded at different anti FGF-2/FGF-2 incubation times—15 min (A1), 30 min (A2), 45 min (A3), 60 min (A4), and 90 min (A5)—showing the impedance responses.


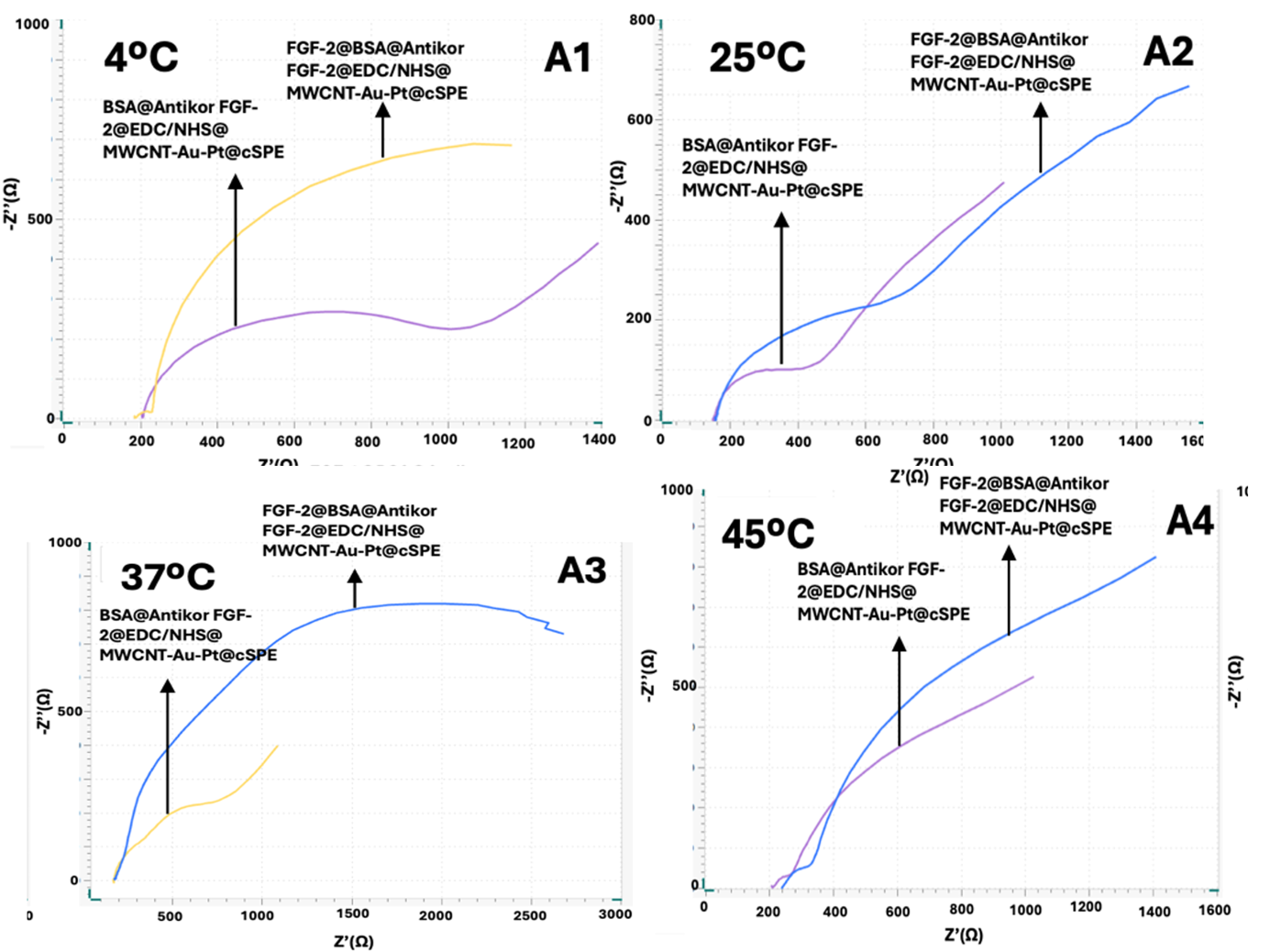


**Figure S6.** Nyquist plots recorded at different anti FGF-2/FGF-2incubation temperatures—4 °C (A1), 25 °C (A2), 37 °C (A3), and 45 °C (A4)—showing the impedance responses.


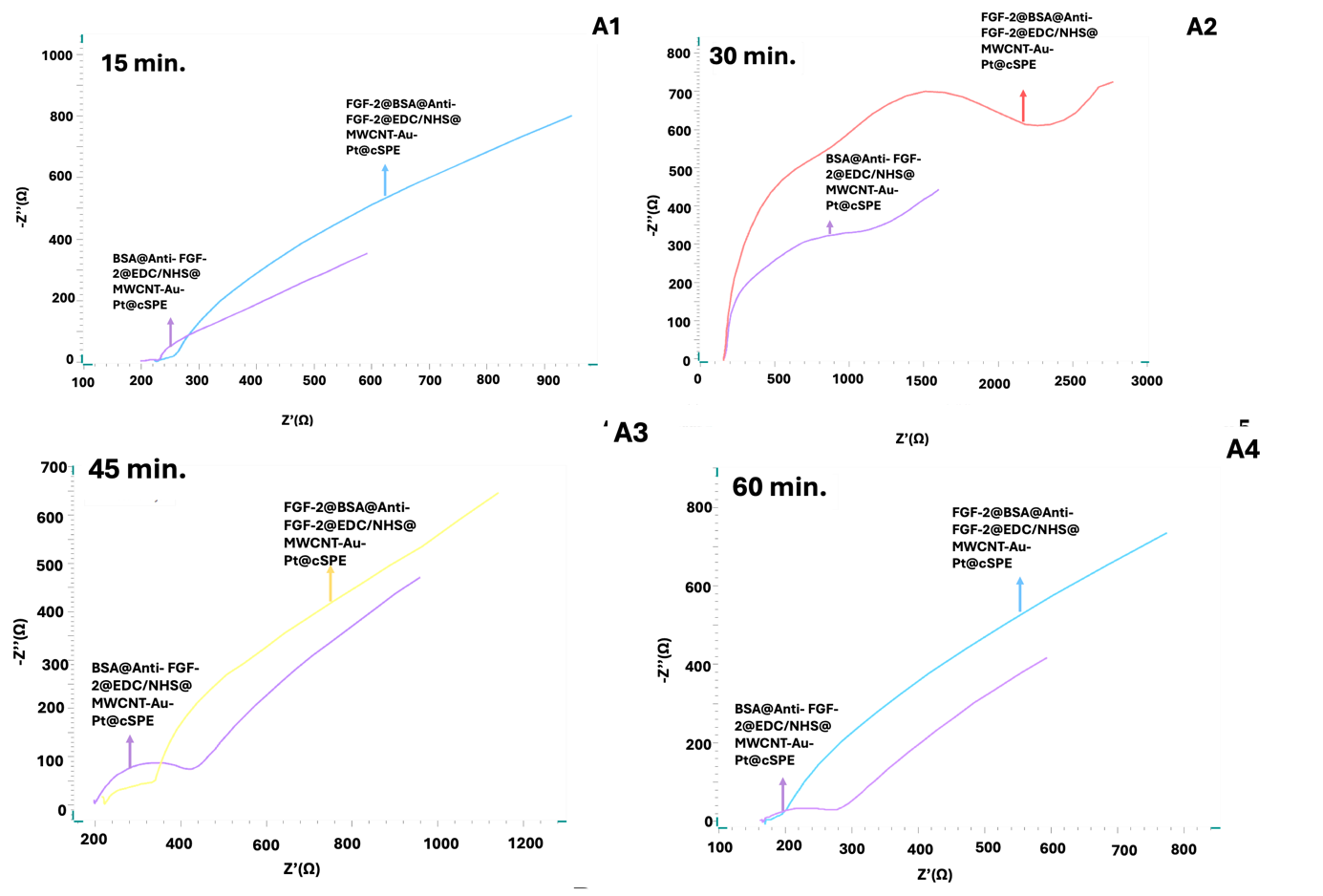


**Figure S7.** Nyquist plots recorded at different anti-FGF-2/FGF-2 incubation times—15 min (A1), 30 min (A2), 45 min (A3), and 60 min (A4)—showing the impedance responses.


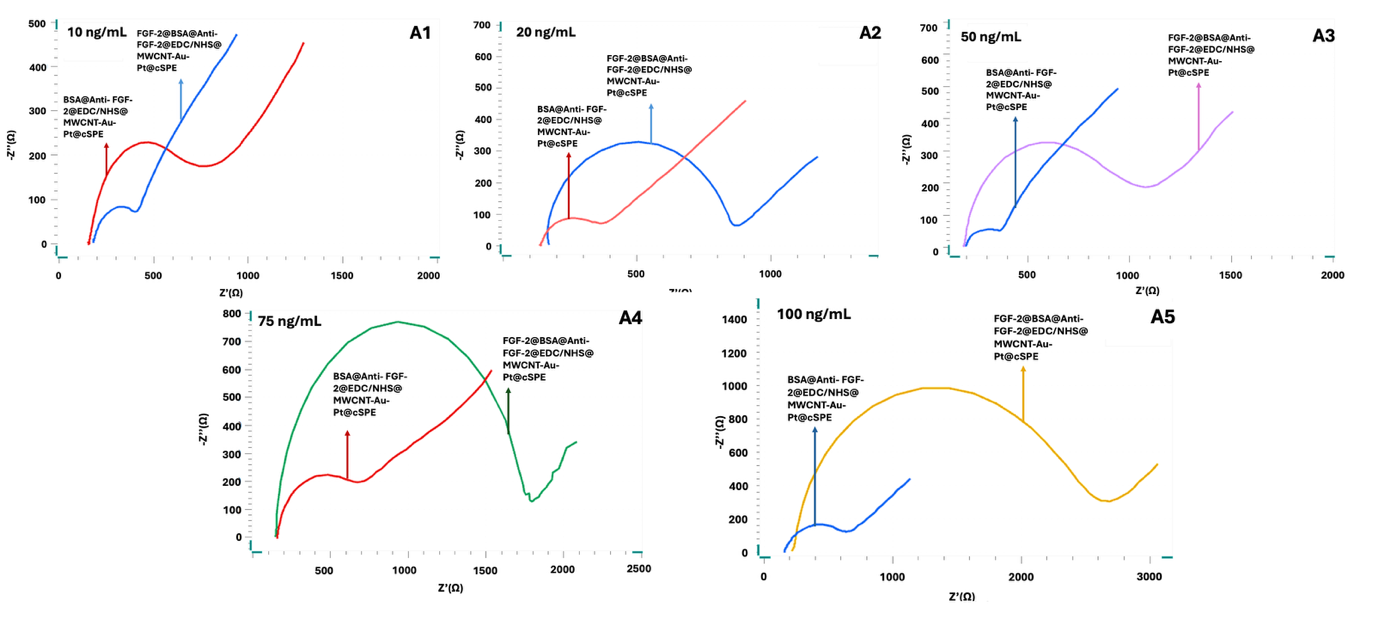


**Figure S8.** Nyquist plots obtained for different FGF-2 concentrations—10 ng/mL (A1), 20 ng/mL (A2), 50 ng/mL (A3), 75 ng/mL (A4), and 100 ng/mL (A5)—showing the impedance responses.
